# Biomechanical evaluation of the proximal chevron osteotomy in comparison to the Lapidus arthrodesis for the correction of hallux valgus deformities

**DOI:** 10.1007/s00264-022-05514-x

**Published:** 2022-07-18

**Authors:** Maximilian F. Kasparek, Emir Benca, Lena Hirtler, Madeleine Willegger, Friedrich Boettner, Shahin Zandieh, Johannes Holinka, Reinhard Windhager, Reinhard Schuh

**Affiliations:** 1 Department of Orthopedics and Trauma Surgery, Medical University of Vienna, Waehringer Guertel 18-20, 1090 Vienna, Austria; 2 Center of Anatomy and Cell Biology, Medical University of Vienna, Waehringer Strasse 13, 1090 Vienna, Austria; 3 Adult Reconstruction & Joint Replacement Division, Hospital for Special Surgery, 535 East 70th Street, New York, NY 10021 USA; 4 Department of Radiology, Hanusch Krankenhaus, Heinrich-Collin-Strasse 20, 1140 Vienna, Austria

**Keywords:** Hallux valgus, Proximal chevron osteotomy, Modified Lapidus arthrodesis, Locking plate, Biomechanical testing

## Abstract

**Purpose:**

The proximal chevron osteotomy and the modified Lapidus arthrodesis are both procedures utilized for deformity correction in patients with severe symptomatic hallux valgus. The aim of the current study was to compare their biomechanical stability when using locking plate fixation.

**Methods:**

Twelve matched pairs of human anatomical lower leg specimens underwent on one side a proximal chevron osteotomy with a medial locking plate and on the other side a modified Lapidus arthrodesis with a plantar locking plate utilizing an interfragmentary compression screw. All specimens underwent bone mineral density (BMD) assessment and were tested in a servohydraulic load frame which applied a load on the centre of the metatarsal head over 1000 loading cycles with subsequently ultimate load testing. Displacement of the proximal and distal bone segment, ultimate load, and bending stiffness were analyzed.

**Results:**

Mean displacement of both procedures showed no statistically significant difference throughout all the loading cycles (0.213 ≤ *p* ≤ 0.834). The mean ultimate load of the proximal chevron osteotomy was 227.9 N (± 232.4) and of the modified Lapidus arthrodesis 162.9 N (± 74.6) (*p* = 0.754). The proximal chevron osteotomy (38.2 N/mm (± 24.9)) had a significantly higher bending stiffness compared to the modified Lapidus arthrodesis (17.3 N/mm (± 9.9)) (*p* = 0.009). There was no correlation between BMD and displacement in all loading cycles, ultimate load, and bending stiffness of either procedure (*p* > 0.05).

**Conclusion:**

Although the bending stiffness of the chevron osteotomy was higher, there was no statistically significant difference between the surgical techniques in mean displacement and ultimate load. The BMD did not influence the overall stability of either reconstruction. Locking plate fixation increases the clinical value of the modified Lapidus arthrodesis by outweighing most of the biomechanical disadvantages in comparison to the proximal chevron osteotomy.

## Introduction

The proximal chevron osteotomy is a proximal V-shaped metatarsal osteotomy that uses an opening wedge principle with a lateral translation of the distal metatarsal fragment [1–3]. In contrast, the modified Lapidus arthrodesis is an arthrodesis of the first tarsometatarsal (TMT) joint [4–7]. Both procedures are used for the correction of severe symptomatic hallux valgus deformities, due to their high potential for angular correction [8, 9]. The disadvantages of these surgical procedures include specific complications, such as osseous nonunion, dorsal elevation malunion, or post-operative loss of correction, which are related to high loads acting on the osteosynthesis [8, 9]. A primary rigid osteosynthesis is recommended to prevent these complications. Historically, the proximal chevron osteotomy was fixed with a single screw [10], compared to two crossed screws for the modified Lapidus arthrodesis [11]. In recent years, locking plates have been introduced for the fixation in these procedures, due to their biomechanical advantages. A superior stability of locking plate fixation as compared to a single cancellous screw fixation, especially in poor bone quality, has been reported for the proximal chevron osteotomy [3]. Similarly, a medial locking plate fixation with a compression screw has been shown to improve cyclic loading testing results, compared to fixation with two screws [12]. In a further biomechanical investigation that compared a compression screw in combination with a plantar locking plate to a dorsomedial locking plate, Klos et al. [13] reported that the plantar plate was significantly stiffer, had less movement at the fused joint space, and tolerated a higher ultimate load.

For the correction of severe hallux valgus deformities, the modified Lapidus arthrodesis provides a potential for higher angular correction, compared to the proximal chevron osteotomy [8, 9]. However, from a biomechanical view, the modified Lapidus arthrodesis has the disadvantage of higher lever forces acting on the osteosynthesis without the inherent stability provided by the V-shaped chevron osteotomy. Locking plate fixation might compensate for these biomechanical shortcomings.

The current human anatomical specimens study compares the biomechanical stability of a proximal chevron osteotomy with a locking plate fixation with the modified Lapidus arthrodesis with a plantar locking plate, with a special focus on the impact of bone quality on the stability of fixation.

## Material and methods

### Specimens

Twelve matched pairs of fresh-frozen human anatomic lower leg specimens, obtained through voluntary body donations for teaching and scientific purposes to the Center of Anatomy and Cell Biology at the Medical University of Vienna, were used in the current study. Institutional review board approval was obtained prior to the study (EK 1856/2014). The ages of the donors ranged between 61 and 99 years (80 years (± 13)), and only specimens without evidence of foot and ankle pathologies as well as any history of forefoot surgery were included. The bone mineral density (BMD) of each specimen was assessed in the calcaneus with dual-energy X-ray absorptiometry (DEXA, GE Lunar Prodigy; GE Healthcare, Chicago, IL, USA), as previously reported by Benca et al. [14] and Willegger et al. [15]. All specimens were frozen and stored at − 80 °C. They were thawed at room temperature for 24 hours before testing to prevent tissue dehydration and changes in biomechanical properties. The soft tissue around the medial cuneiform bone and first metatarsal was completely removed and all specimens were visually inspected to confirm intact bone structure. During the surgical preparation, the specimens were moistened with 0.9% saline solution to prevent desiccation. In a randomized order, a proximal chevron osteotomy was performed on one specimen and a modified Lapidus arthrodesis on the contralateral specimen of the same pair. Both groups contained an equal number of left and right metatarsals.

### Surgical technique—proximal chevron osteotomy

**Fig. 1:**
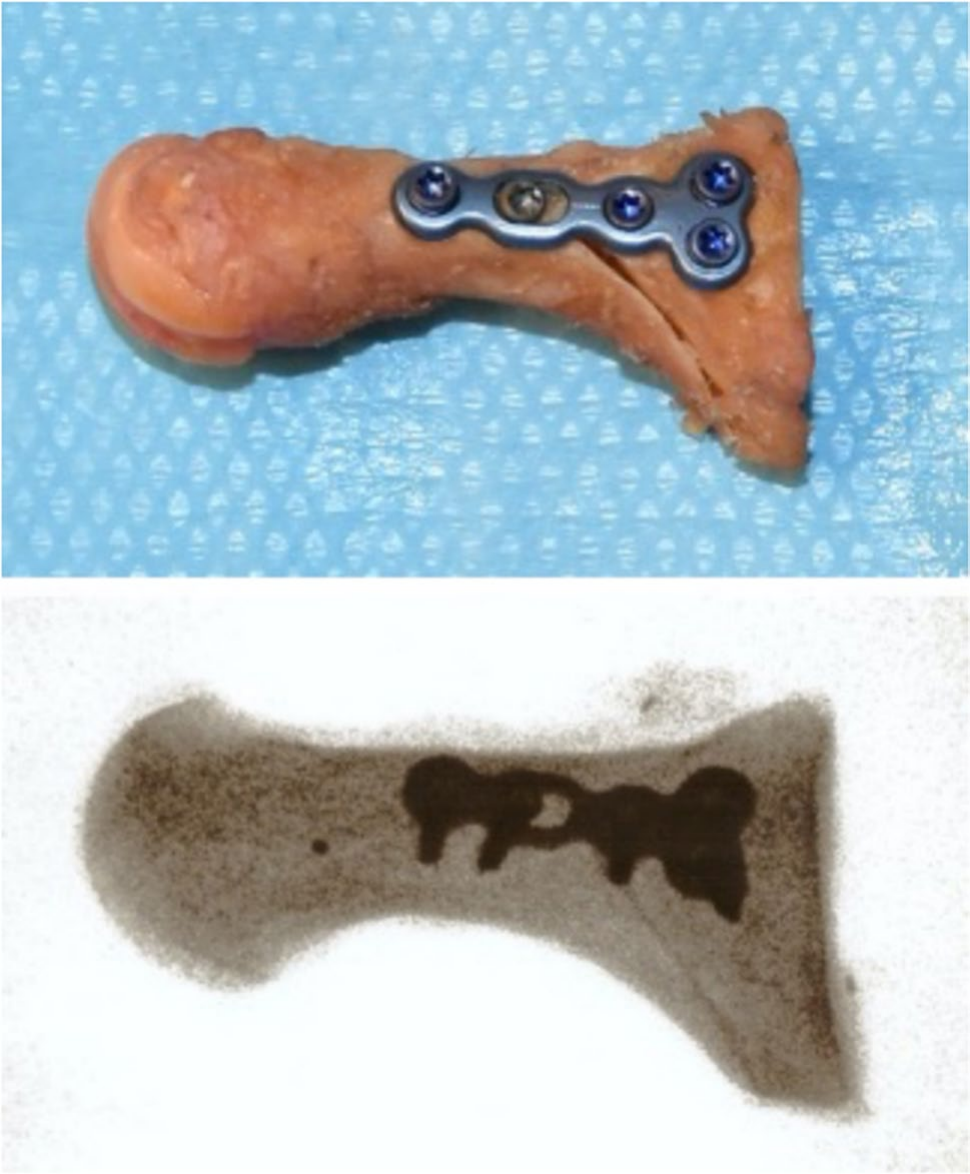
Post-operative photograph and fluoroscopy image of a proximal chevron osteotomy fixed with a variable medial locking plate (3 Hole Low Profile T-Plate 2,4 mm, Arthrex Inc., Naples FL, USA)

The apex of the osteotomy was located 20 mm distal to the proximal metatarsocuneiform joint surface and in the centre of the metatarsal shaft. Using a microsagittal saw, a proximal-based chevron osteotomy at a 90° angle was performed. After confirmation of the complete bony release of the first metatarsal, the distal fragment was translated 3 mm laterally and angulated 10° laterally for simulation of deformity correction. The simulated deformity correction was temporally fixed with a Kirschner wire in the direction from the dorsal part of the distal fragment to the plantar part of the proximal metatarsal base. The overhanging prominent part of the proximal fragment was trimmed to be on one level with the distal fragment. A locking plate was used (3 Hole Low Profile T-Plate 2.4 mm, Arthrex Inc., Naples FL, USA) for the fixation of the osteotomy, and it was fixed with a bicortical non-locking screw. Bicortical locking screws were inserted in all three proximal screw holes of the T-plate and one distal screw hole, resulting in a rigid fixed-angle osteosynthesis. After the plate fixation was complete, the K-wire was removed (Fig. 1).

### Surgical technique—modified Lapidus arthrodesis

**Fig. 2:**
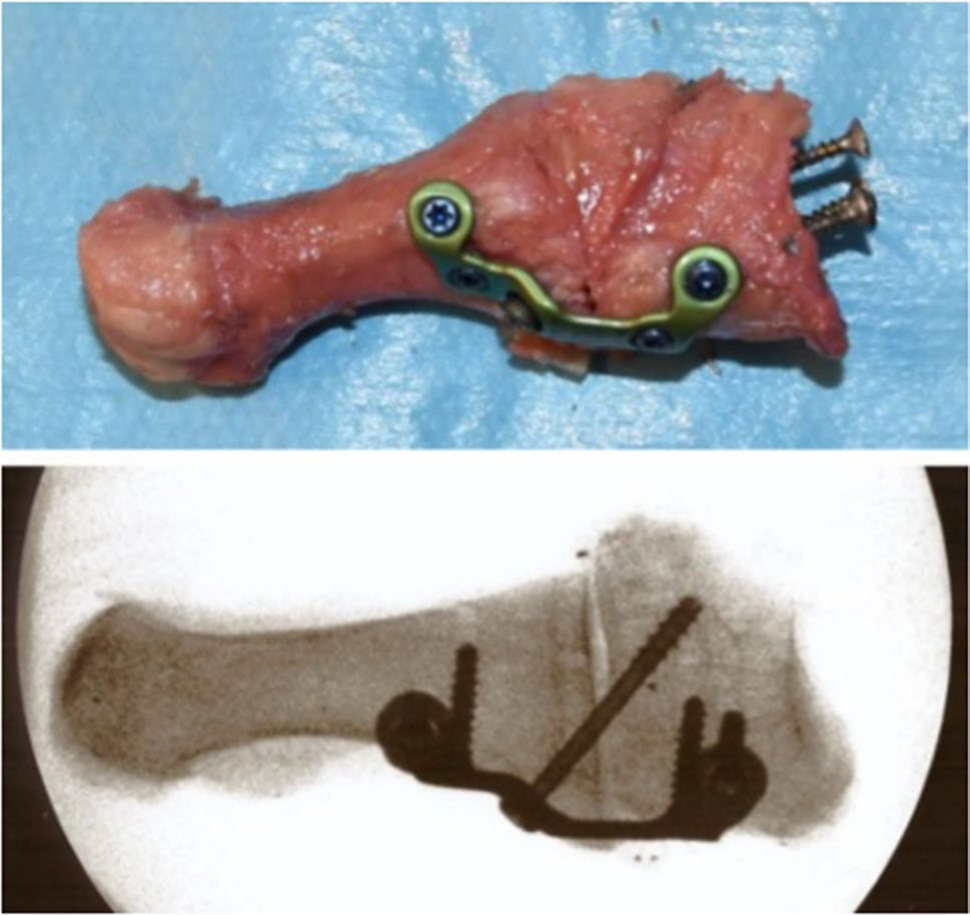
Post-operative photograph and fluoroscopy image of a modified Lapidus arthrodesis fixed with a plantar plate with a compression screw (Plantar Lapidus Plate, Arthrex Inc., Naples FL, USA). For fixation to the steel cups with Wood’s metal, three conventional wood screws were inserted into the proximal base of the medial cuneiform bone or the first metatarsal in all specimens

The TMT joint was exposed, and it was verified that the joint capsule was removed. The cartilage of the TMT joint was removed through a biplanar wedge resection to simulate deformity correction, using an oscillating bone saw. The joint was temporarily fixed with two Kirschner wires. The locking plate (Plantar Lapidus Plate, Arthrex Inc., Naples FL, USA) was placed on the plantar side of the TMT joint. The plates were precontoured for the right or the left side. In the first step, the plate was fixed distally with two 3.5 mm locking screws. The hole for the compression screw was drilled with the aid of the aiming guide, and a 4.0 mm cannulated compression screw was inserted. Before the cannulated compression screw was finally tightened to achieve compression between the medial cuneiform bone and first metatarsal bone, the two Kirschner wires were removed. Finally, two locking screws, which are specially designed to not interfere with the compression screw, were inserted into the proximal holes of the plate (Fig. 2).

### Biomechanical testing

**Fig. 3:**
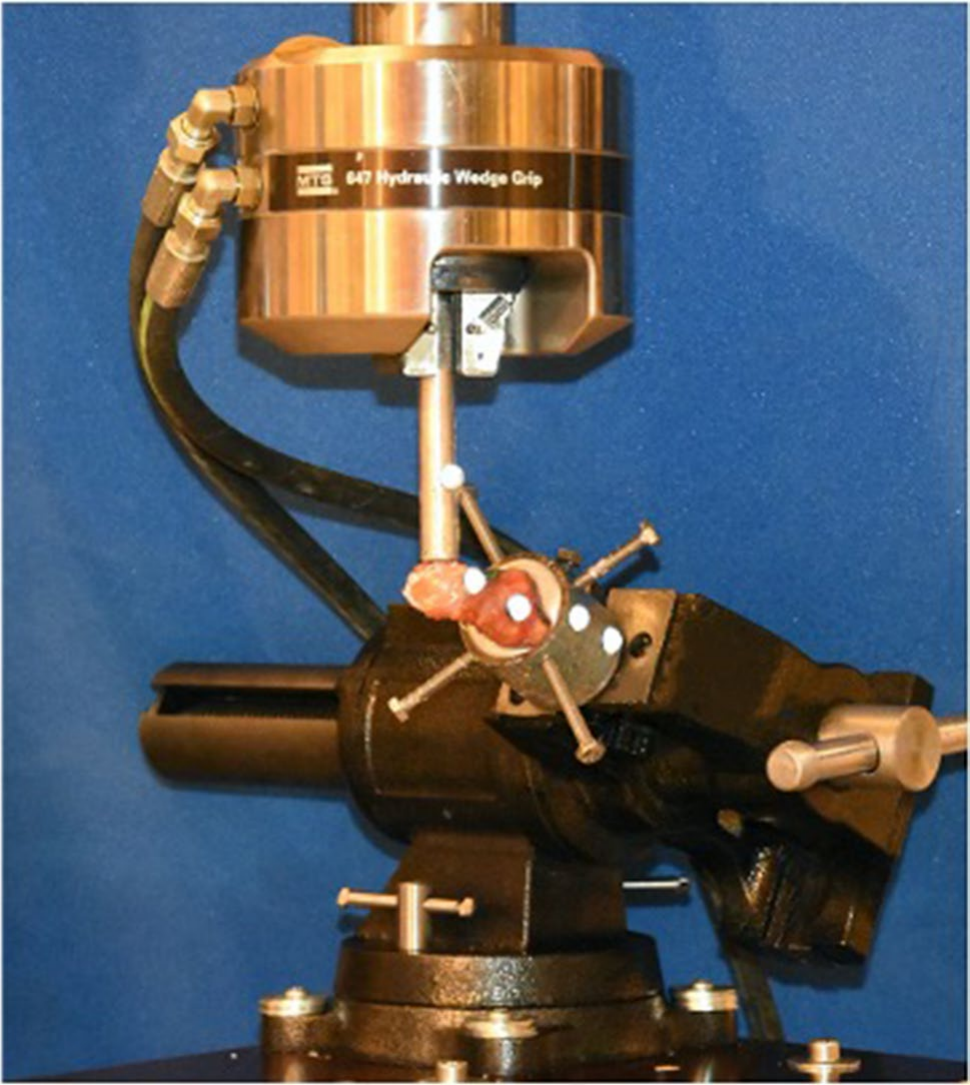
The biomechanical test setup. All specimens were embedded in steel cups with Wood’s metal and fixed into a machine vice. The servohydraulic load frame (858 Mini Bionix®, MTS® Systems Corporation, Eden Prairie, MN, USA) loaded the first metatarsal head of the specimens over a metal stamp in a plantar-to-dorsal direction. Five hemispherical markers (two markers on the steel cup, two markers on the first metatarsal, and one on the metal stamp of the loading frame) were fixed to the specimens for the kinematic measurements, using an opto-electronic motion capture system (Smart-E; BTS Bioengineering, Milan, Italy)

All specimens were embedded in steel cups with a 40 mm diameter with the use of Wood’s metal. To ensure a stable fixation in the cups, three conventional wood screws were inserted into the proximal base of the first metatarsal or medial cuneiform bone. The steel cups were fixed to a servohydraulic load frame (858 Mini Bionix, MTS Systems Corporation, Eden Prairie, MN, USA) (Fig. 3). A force measurement transducer that was integrated into the load frame continuously recorded the load, with a sampling frequency of 60 Hz. This system has a low uncertainly of 1% for the measurement of force. The kinematic measurements were performed with an opto-electronic motion capture system (Smart-E; BTS Bioengineering, Milan, Italy), by means of four IR (infrared) cameras and reflecting markers with a sampling rate of 120 Hz during the loading process, and five hemispherical markers with a 10 mm diameter that were fixed to the steel cup with epoxy glue (2 markers), distal part of the first metatarsal (2 markers), and the actuator of the loading frame (1 marker). Videos were recorded of all the tests with a conventional camera (D7200; Nikon, Tokyo, Japan).

All specimens were tested in a biomechanical setup to simulate post-operative in vivo load conditions. The actuator of the servohydraulic load frame applied a cyclic load with an amplitude of 31 N in a plantar-to-dorsal direction at the center of the distal metatarsal head. A peak load of 31 N was used for the testing of the fixation techniques of first metatarsal osteotomies, and it represents one-third of the load of the mean Ludloff osteotomy fixation failure, based on the results of Trnka et al. [16]. The specimens underwent 1000 loading cycles, unless a major construct fixation failure (distal fragment dorsal angulation greater than 10°) occurred. It was defined that if a construct failed before 1000 loading cycles were conducted, the load applied at this cycle was determined as the load to failure for this specimen. Displacement of the metatarsal head was measured in millimeter (mm) for the following loading cycles: 1, 10, 50, 100, 200, 300, 400, 500, 600, 700, 800, 900, and 1000. These cycles were linear ramps with a gradient of 7.75 N/s and a corresponding peak load of 31 N, obtained in 4 s. The loading cycles between these defined measurement cycles were conducted as cyclic loads at 0.5 Hz. After completion of the 1000 cycles, the ultimate load test was performed by increasing the load until the constructs failed. Bending stiffness of the construct (slope of the load–displacement curve (N/mm)) was measured in the ascending linear region of the load–displacement curve of the ultimate load test [14]. After completion of the biomechanical testing protocol, all specimens were extracted from the metal cup, inspected for the mode of failure, and photographed for documentation.

### Statistical analysis

Values were reported as mean (± standard deviation). The Kolmogorov–Smirnov test was used to test normal distribution. For normally distributed variables, the paired *t*-test or unpaired *t*-test was used, and, for non-normal distributed variables, the paired, two-tailed Wilcoxon rank test was used for statistical comparison of BMD, displacement, bending stiffness, and the ultimate load between the two fixation groups. Pearson’s correlation coefficient was used to investigate correlations between BMD, displacement, and ultimate load. All statistical tests were performed using the SPSS Version 26.0 for Mac OS X (IBM Corp., Armonk, NY, USA) and GraphPad Prism® Version 8.0 for Mac OS X (GraphPad Software Inc.; La Jolla, CA, USA).

## Results

### Displacement

**Fig. 4:**
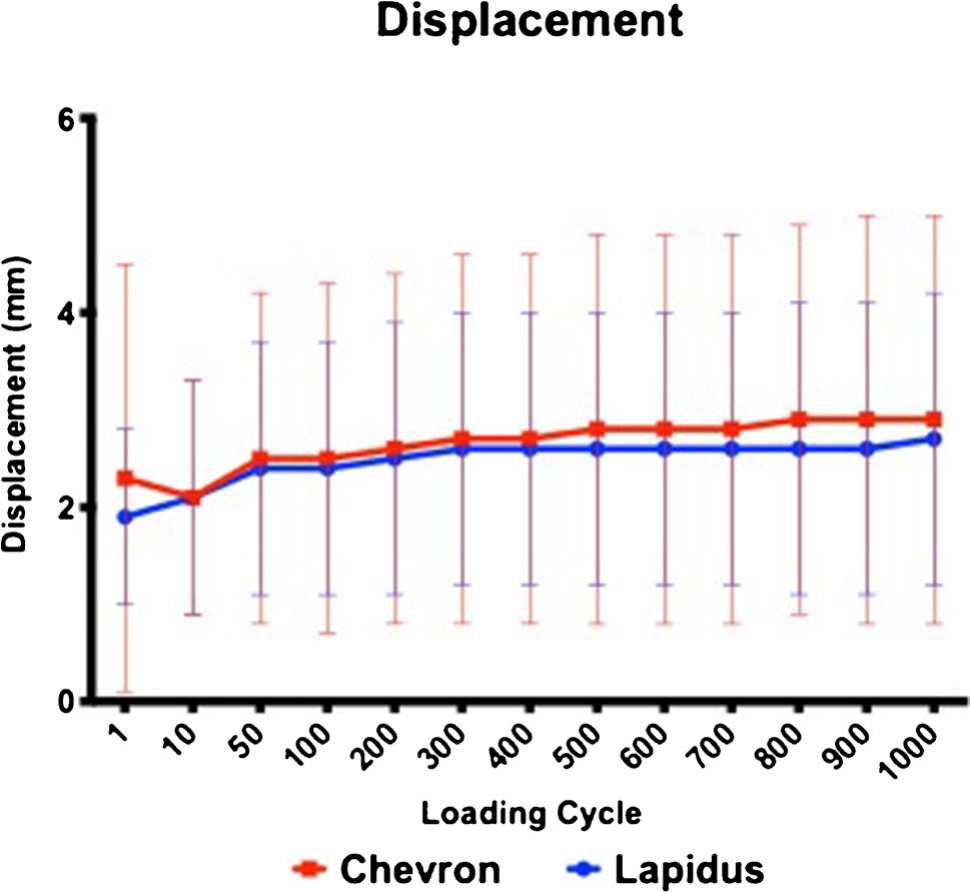
Displacement in the proximal chevron osteotomy (red) and the modified Lapidus arthrodesis (blue) was comparable over all loading cycles

The mean displacement in the proximal chevron osteotomy (2.3 mm (± 2.2)) and modified Lapidus arthrodesis (1.9 mm (± 1.0)) was not statistically significantly different at the first loading cycle (*p* = 0.638). Mean displacement increased progressively in both groups over all loading cycles. At the 1000th loading cycle, the mean displacement was 2.9 mm (± 2.2) in the proximal chevron group and 2.7 mm (± 1.5) in the modified Lapidus group (*p* = 0.213) (Table 1 and Fig. 4).

**Table 1:** Displacement in the proximal chevron osteotomy group and the modified Lapidus arthrodesis group in all loading cycles

		Proximal chevron osteotomy		Modified Lapidus arthrodesis		
Cycles	No	Mean (mm)	SD	Mean (mm)	SD	*p*-value
1	12	2.3	2.2	1.9	0.9	0.638
10	11	2.1	1.2	2.1	1.2	0.834
50	11	2.5	1.7	2.4	1.3	0.798
100	11	2.5	1.8	2.4	1.3	0.727
200	11	2.6	1.8	2.5	1.4	0.680
300	11	2.7	1.9	2.6	1.4	0.743
400	11	2.7	1.9	2.6	1.4	0.739
500	11	2.8	2.0	2.6	1.4	0.333
600	11	2.8	2.0	2.6	1.4	0.286
700	11	2.8	2.0	2.6	1.4	0.286
800	11	2.9	2.0	2.6	1.5	0.248
900	11	2.9	2.1	2.6	1.5	0.248
1,000	11	2.9	2.1	2.7	1.5	0.213

*No.*, number of pairs; *SD*, standard deviation

### Ultimate load testing and bending stiffness

The proximal chevron osteotomy had a mean ultimate load of 227.9 N (± 232.4) and the modified Lapidus arthrodesis 162.9 N (± 74.6) (*p* = 0.754). The mean bending stiffness in the ultimate load test was significantly higher in the proximal chevron group (38.2 N/mm (± 24.9)) compared to the modified Lapidus group (17.3 N/mm (± 9.9) (*p* = 0.009).

### Mode of failure

**Fig. 5:**
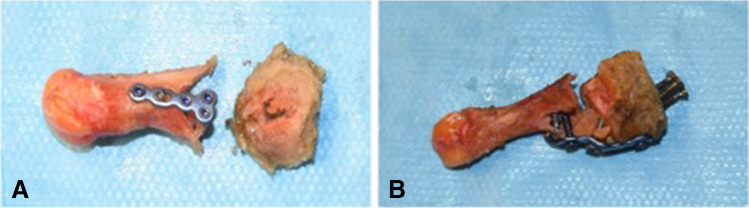
The most common failure mode for the proximal chevron was the proximal locking screws pulling out (**A**) and, for the modified arthrodesis, fracture of the proximal part of first metatarsal (**B**)

One proximal chevron construct failed between the first and 10^th^ loading cycles. All the other constructs completed all 1000 loading cycles. The most common failure mode for the proximal chevron constructs was the proximal locking screws pulling out (10 constructs) and medial deviation of the plate (two constructs). For the modified Lapidus arthrodesis constructs, the most common failure mechanism was the failure of the distal fixation due to the distal locking screws pulling out or proximal fracture of the first metatarsal (11 constructs) and the proximal locking screws pulling out (1 construct) (Fig. 5).

### Bone mineral density

The mean BMD was not statistically significantly different between the proximal chevron group 0.488 g/cm^2^ (± 0.260) and modified Lapidus group 0.469 g/cm^2^ (± 0.215) (*p* = 0.570). There was no correlation between BMD and age in overall (*R* =  − 0.178, *p* = 0.404), as well as in the proximal chevron group (*R* =  − 0.085, *p* = 0.792) and in the modified Lapidus group (*R* =  − 0.293, *p* = 0.356). The mean BMD was significantly higher in male specimens (0.547 g/cm^2^ (± 0.225) compared to female specimens (0.273 g/cm^2^ (± 0.109) (*p* = 0.009). The BMD did not correlate with displacement in all loading cycles in the proximal chevron group (− 0.462 ≤ *R* ≤ 0.300, 0.131 ≤ *p* ≤ 0.509) and the modified Lapidus group (− 0.167 ≤ *R* ≤  − 0.078, 0.603 ≤ *p* ≤ 0.809). There was also no correlation between the BMD and the ultimate load in the proximal chevron group (*R* = 0.108, *p* = 0.739) and the modified Lapidus group (*R* = 0.268, *p* = 0.400) or with the pooled ultimate load (*R* = 0.137, *p* = 0.524). Moreover, the BMD did not correlate with the pooled bending stiffness (*R* = 0.258, *p* = 0.236) in both groups (proximal chevron (*R* = 0.160, *p* = 0.639), modified Lapidus (*R* = 0.421, *p* = 0.173)).

## Discussion

The current study is the first to compare the biomechanical behavior of a proximal metatarsal osteotomy and a TMT arthrodesis for the correction of severe hallux valgus deformities, using locking plate fixation. It was found that the mean displacement in all loading cycles was comparable in the paired analysis. Furthermore, the mean ultimate load was not statistically significantly different between the two procedures. The mean bending stiffness of the proximal chevron osteotomy was significantly higher than for the modified Lapidus arthrodesis. The biomechanical stability of both correction techniques was influenced by the bone mineral density.

In the current study, both procedures were tested in a cyclic loading test setup to simulate post-operative in vivo load conditions. The mean displacements of the proximal chevron osteotomy (2.3 (± 2.2 mm)) and modified Lapidus arthrodesis (1.9 mm (± 1.0)) were comparable at the first loading cycle. The mean displacement increased slightly in both groups. At the 1000th loading cycle (proximal chevron osteotomy 2.9 mm (± 2.2) group, modified Lapidus 2.7 mm (± 1.5)), there were no clinically significant differences in the mean displacement. Scott et al. [17] and Schuh et al. [3] previously reported an increase in the dorsal angulation with an increasing number of dorsal loading cycles for the proximal chevron osteotomy using locking plate fixation. The data of the mean displacement in the modified Lapidus arthrodesis align with the reported data from Klos et al. [13], who reported a displacement of 1.5 mm (± 0.7) with a compression screw and a plantar locking plate in a biomechanical test setup.

In the ultimate load testing, no statistically significant difference was found between the mean ultimate load of 227.9 N (± 232.4) for the proximal chevron osteotomy in comparison to 162.9 N (± 74.6) for the modified Lapidus arthrodesis. In a prior biomechanical study by Schuh et al. [3], they found that a lateral angle-stable plate demonstrated superior fixation stability in proximal chevron osteotomy compared to fixation with a single screw. They reported comparable data from the ultimate load test (220.9 N (± 202.5)). Moreover, the results of the modified Lapidus arthrodesis are similar to previously reported biomechanical data from Klos et al. [13], who reported an ultimate load of 192.6 N (± 26.8) for a plantar angle-stable plate in combination with a compression screw inserted in distal-dorsal to proximal-plantar direction. In a biomechanical study, Cottom and Baker [18] tested the same plate that was used in the current study. They did not perform a cyclic load testing before ultimate load-to-failure testing, but, nevertheless, reported a comparable mean ultimate load (197.5 N (± 108.6)). In the current study, the proximal chevron osteotomy had a significantly higher mean bending stiffness (38.2 N/mm (± 24.9)) compared to the modified Lapidus group (17.3 N/mm (± 9.9)), which might be related to the fact that the proximal chevron osteotomy has to withstand lower lever forces and the bony overlap of the proximal and distal osteotomy parts provide an additional inherent stability. Moreover, the mode-of-failure analysis revealed different locations of failure in the constructs. Whereas the most common failure mode of the proximal chevron constructs was the proximal locking screws pulling out, the modified Lapidus arthrodesis constructs failed at the distal plate fixation. One proximal chevron construct, which had the lowest BMD of all the specimens, failed between the first and the 10th loading cycle. Nevertheless, the BMD was comparable between the groups, and both groups with the use of locking plates did not correlate with BMD.

The literature on the correction of severe hallux valgus deformities describes a wide variation of surgical procedures, including proximal metatarsal osteotomies and the modified Lapidus arthrodesis [19, 20]. Haas et al. [21] compared a first metatarsal closing base wedge osteotomy and the modified Lapidus arthrodesis for correction of a moderate to severe hallux valgus deformity. Both procedures proved to be effective for the correction of these deformities; however, the modified Lapidus provided superior results in maintaining the intermetatarsal angular correction. In addition, data from a meta-analysis by Willegger et al. [9] revealed that the first tarsometatarsal joint arthrodesis has the highest potential for an angular correction in hallux valgus deformity correction. Moreover, these results support the mathematical principle that the potential for angular correction increases from distal to proximal. However, concomitant with the potential for higher angular correction of the first tarsometatarsal joint arthrodesis, the lever arm forces acting on the osteosynthesis increase. The current study was the first to compare the biomechanical behavior of a proximal metatarsal osteotomy and the modified Lapidus arthrodesis. It was revealed that, despite the higher lever arm forces acting on the osteosynthesis of the modified Lapidus arthrodesis in comparison to the proximal chevron osteotomy, the mean displacement and mean ultimate load were not statistically significantly different with the utilization of a locking plate fixation. However, for the construct bending stiffness, the proximal chevron osteotomy had superior results. The data of the current study show that the utilization of a locking plate fixation for the modified Lapidus arthrodesis compensates for most biomechanical disadvantages, compared to the proximal chevron osteotomy. In the future, randomized prospective high-quality clinical trials are needed to analyze if the proximal chevron osteotomy or the modified Lapidus arthrodesis with a locking plate fixation will prove to be advantageous in the treatment of severe hallux valgus deformities.

The current study has the following limitations. First, the biomechanical setup does not reproduce physiologic conditions completely since the medial cuneiform bone was resected in the proximal chevron osteotomy group. However, the biomechanical setting of the proximal chevron osteotomy is similar to that of Schuh et al. [3], who also resected the medial cuneiform bone. Moreover, we aimed to compare the biomechanical stability of the osteosynthesis, and preserving the TMT joint in the proximal chevron osteotomy group might have influenced the biomechanical results. Second, the data were generated in a biomechanical experimental test setup, and all tests were performed under controlled conditions. The utilized specimens did not reflect patients with a severe symptomatic hallux valgus deformity. Moreover, the surrounding soft tissue was removed, resulting in a more unstable scenario compared to in vivo conditions. Therefore, the data of the current study cannot be directly transferred to clinical use. Third, the sample size was small and the mean donor age was high. However, small sample sizes are a common weakness of biomechanical studies because of the limited availability of human specimens. The mean donor age was comparable to that in other biomechanical studies of the proximal chevron osteotomy or modified Lapidus arthrodesis [3, 13]. Due to the high donor age, the BMD and the overall bone tissue quality might be lower than in most patients undergoing angular correction of the first ray. Therefore, the data of the current study might represent a high-risk patient collective. Despite the high-risk scenario, both procedures with locking plates were independent of BMD.

## Conclusions

The proximal chevron osteotomy with a variable medial locking plate and the modified Lapidus arthrodesis with a plantar locking plate with an interfragmentary compression screw through the plate had a comparable mean displacement under cyclic loading and mean ultimate load to failure. The bending stiffness of the proximal chevron osteotomy was higher than that of the modified Lapidus arthrodesis. The quality of the bone had no impact on the biomechanical stability of either procedure. For daily clinical practice, the data from the current study show that locking plate fixation mostly outweighs the biomechanical disadvantages of the modified Lapidus arthrodesis in comparison to the proximal chevron osteotomy. The current findings increase the clinical value of the modified Lapidus arthrodesis, which proved to be the surgical procedure with the highest potential for an angular correction in hallux valgus surgery.

## Data Availability

The data of the current study are available from the corresponding author upon reasonable request.
